# LUM Expression and Its Prognostic Significance in Gastric Cancer

**DOI:** 10.3389/fonc.2020.00605

**Published:** 2020-05-15

**Authors:** Xiaowei Chen, Xin Li, Xueju Hu, Fei Jiang, Yan Shen, Rui Xu, Leilei Wu, Pingmin Wei, Xiaobing Shen

**Affiliations:** ^1^Key Laboratory of Environmental Medicine Engineering, Ministry of Education, School of Public Health, Southeast University, Nanjing, China; ^2^Department of Epidemiology and Health Statistics, School of Public Health, Southeast University, Nanjing, China

**Keywords:** gastric cancer, LUM, lumican, TCGA, GSEA, prognosis

## Abstract

**Background:** Lumican (LUM) is a member of the small leucine-rich proteoglycan family and plays dual roles as an oncogene and a tumor suppressor gene. The effect of LUM on tumors is still controversial.

**Methods:** Gene expression profiles and clinical data of gastric cancer (GC) were downloaded from The Cancer Genome Atlas (TCGA) database. The expression difference of LUM in GC tissues and adjacent nontumor tissues was analyzed by R software and verified by quantitative real-time polymerase chain reaction (qRT-PCR) and comprehensive meta-analysis. The relationship between LUM expression and clinicopathological parameters was assessed by chi-square test and logistic regression. Kaplan–Meier survival analysis and Cox proportional hazards regression model were chosen to assess the effect of LUM expression on survival. Gene set enrichment analysis (GSEA) was used to screen the signaling pathways involved in GC between the low and the high LUM expression datasets.

**Results:** The expression of LUM in GC tissues was significantly higher than that in adjacent nontumor tissues (*P* < 0.001) from the TCGA database. qRT-PCR (*P* = 0.022) and comprehensive meta-analysis (standard mean difference = 0.90, 95% CI: 0.34–1.46) demonstrated that LUM was upregulated in GC. The chi-square test showed that the high expression of LUM was correlated with tumor differentiation (*P* = 0.024) and T stage (*P* = 0.004). Logistic regression analysis showed that high LUM expression was significantly correlated with tumor differentiation (OR = 1.543 for poor vs. well or moderate, *P* = 0.043), pathological stage (OR = 3.149 for stage II vs. stage I, *P* = 0.001; OR = 2.505 for stage III vs. stage I, *P* = 0.007), and T classification (OR = 13.304 for T2 vs. T1, *P* = 0.014; OR = 18.434 for T3 vs. T1, *P* = 0.005; OR = 30.649 for T4 vs. T1, *P* = 0.001). The Kaplan–Meier curves suggested that patients with high LUM expression had a poor prognosis. Multivariate analysis showed that a high expression of LUM was an important independent predictor of poor overall survival (HR, 1.189; 95% CI, 1.011–1.400; *P* = 0.037). GSEA indicated that 14 signaling pathways were evidently enriched in samples with the high-LUM expression phenotype.

**Conclusions:** LUM might act as an oncogene in the progression of GC and could be regarded as a potential prognostic indicator and therapeutic target for GC.

## Introduction

Gastric cancer (GC) is one of the most common malignant tumors in the world. According to the GLOBOCAN 2018 estimation, there were 1.0337 million new cases of gastric cancer worldwide, accounting for 5.7% of the total, making GC the fifth most common malignant tumor. Approximately 783,000 of these patients died of gastric cancer, accounting for 8.2% of the total GC cases. Thus, GC is considered as the third leading cause of cancer-related deaths worldwide (1). As GC symptoms often present in the later stages only, many patients with GC have advanced stage disease when given a definitive diagnosis. Despite advancements in treatment, the clinical outcomes of patients with advanced GC are still poor. Therefore, it is crucial to identify a sensitive and specific biomarker that could predict the prognosis of GC and serve as a target for GC treatment.

Small leucine-rich proteoglycan (SLRP), a subtype of extracellular matrix (ECM) proteoglycan, is a signal molecule involved in a variety of intercellular activities. It not only has vital functions for regulating extracellular water balance and collagen fiber formation but also exerts a great influence on tumor growth, adhesion, and migration (2–4). SLRP consists of 17 members, including decorin, biglycan, lumican, and so on, which can be further categorized into five distinct classes based on their evolutionary protein conservation, leucine rich repeats, N-terminal cysteine-rich clusters, and chromosomal organization (5, 6). Decorin belongs to class I SLRP family and consists of a protein core containing leucine repeats with a glycosaminoglycan (GAG) chain consisting of either chondroitin sulfate (CS) or dermatan sulfate (DS) (7). The GAG chain is tissue-specific, with DS in tendon and skin and CS in bone and cartilage (8). Current studies have presented two mechanisms on the anti-tumor capacity of decorin, of which one is directly inhibiting the signal transduction pathways in tumor cells via interaction with two receptor tyrosine kinases, Met and EGFR (9, 10), and the other is inducing autophagy to reduce the metastasis and spread of cancer cells (11). Biglycan, a class I SLRP, consists of a 42-kDa core protein and contains similar structures with decorin in two GAG chains (12). Ubiquitously expressed in ECM, biglycan serves as a key matrix component and an essential signaling molecule (12). Biglycan could initiate inflammation, facilitate cancer cell migration, and alter tumor proliferation (13–16). Lumican (LUM) is a class II SLRP that has a 40-kDa core protein (*7*) located in the 12q21.3-q22 region of the 12th chromosome (17), consisting of three exons and two introns, with a full length of 6.9 kb. It is a keratan sulfate proteoglycan and was first found in the corneal stroma (18). As an important component of the extracellular matrix (19), lumican is widely expressed in various tissues of the human body in the form of proteoglycan (20, 21). It is not only expressed in the intervertebral disc, skin, lung, liver, breast, pancreas, colorectal, skeletal muscle, articular cartilage, and other tissues but also abnormally expressed in a variety of malignant tumors or tumor stroma (22). The process of tumor proliferation, invasion, and migration is accompanied by the synthesis and the degradation of extracellular matrix, abnormal expression of lumican, or its interaction with integrin which can affect the formation of the extracellular matrix, thus affecting tumor metastasis and invasion (23). At the same time, lumican regulates tumor angiogenesis by participating in the formation of the tubular structure of epithelial primordial cells, which in turn affects tumor proliferation (24). The inflammatory state in the tumor microenvironment may destroy the immune function of the body and promote tumor formation, while some studies have shown that lumican is involved in tumor inflammatory signal transduction, which affects the development of tumors by binding to integrin subunits such as β2, α, and αL on polymorphonuclear leukocytes (25). Some studies have also suggested that lumican plays a key biological role in regulating tumor development and dissemination by using a plethora of signaling cascades to regulate intracellular and extracellular signal transduction (26). Lumican has two different effects on the occurrence and the development of tumors. It may act as either an oncogene or a tumor suppressor gene. The effect of lumican on tumors is still controversial, and its positive and negative correlation with tumor invasiveness has been reported (27).

Based on The Cancer Genome Atlas (TCGA) dataset, the Gene Expression Omnibus (GEO) dataset, and quantitative real-time polymerase chain reaction (qRT-PCR), this study explored the relationship between LUM gene expression and the clinicopathological features of patients with GC and its prognostic significance to provide more evidence for its potential role as a prognostic biomarker in GC.

## Materials and Methods

### TCGA Data and Samples

On or before November 11, 2019, the original gene mRNA data for 375 samples of GC tissues and 32 samples of adjacent nontumor tissues were downloaded from the TCGA database (https://portal.gdc.cancer.gov). The clinical data of the GC patients were also obtained from the TCGA database. The details included age, gender, grade, pathological stage, T stage, N stage, M stage, and vital status. The details of the patients are shown in Table 1. Since the data were provided by TCGA, approval from the Ethics Committee was not required. This study was fully in line with the guidelines for the NIH TCGA human subject protection and data access policies. In addition, 47 pairs of GC tissues and adjacent nontumor tissues were collected from Zhongda Hospital, Southeast University, and approved by the Ethics Committee of Zhongda Hospital, Southeast University. All participants signed an informed consent form. These samples were obtained after surgical resection from patients who had never received preoperative radiotherapy or chemotherapy. Then, the samples were saved in RNA later (Ambion, Austin, TX, USA) and stored at −80°C until further use.

**Table 1:** Characteristics of patients with GC.

**Characteristics**	**Variable**	**Patients (375)**	**Percentages (%)**
Age	<65 years	155	41.33
	≥65 years	216	57.60
	Unknown	4	1.07
Gender	Male	241	64.27
	Female	134	35.73
Grade	G1	10	2.67
	G2	137	36.53
	G3	219	58.40
	GX	9	2.40
Pathological stage	I	53	14.13
	II	111	29.60
	III	150	40.00
	IV	38	10.13
	Unknown	23	6.14
T classification	T1	19	5.07
	T2	80	21.33
	T3	168	44.80
	T4	100	26.67
	TX	8	2.13
N classification	N0	111	29.60
	N1	97	25.87
	N2	75	20.00
	N3	74	19.73
	NX	16	4.27
	Unknown	2	0.53
M classification	M0	330	88.00
	M1	25	6.67
	MX	20	5.33
Vital status	Alive	244	65.07
	Death	131	34.93

*GC, gastric cancer. Data are presented as number (%)*.

### LUM Expression Analysis and Survival Analysis

Perl programming language was used to sort and merge the downloaded original gene expression data, and the limma package of R software was used to extract the LUM expression data from the dataset. The limma package and beeswarm package were used to visualize the extracted data and draw scatter difference diagrams. Perl programming language was used to extract survival data from clinical data and remove the data with incomplete survival time and survival status information. After that, the complete survival information was matched with the LUM expression data, and finally, the data of 368 patients who met the requirements were obtained. In accordance with the median expression value, the LUM mRNA expression level was divided into two groups (high-LUM expression group and low-LUM expression group). The survival package of R software was used for visualization, and the Kaplan–Meier survival curve was obtained.

### The Verification of LUM by qRT-PCR

Total RNA was extracted from 47 pairs of GC tissues and adjacent nontumor tissues frozen in liquid nitrogen with TRIzol reagent (Invitrogen, Carlsbad, CA, USA). The concentration and purity of the total RNA was determined by a NanoDrop 2000 spectrophotometer (Thermo Fisher Scientific, USA). The PrimeScript™ RT reagent kit (TAKARA) was used to reverse-transcribe RNA into complementary DNA (cDNA) according to the instructions. The PCR conditions on the StepOnePlus PCR System (Applied Biosystems, Waltham, MA, USA) with 2x RealStar Power SYBR Mixture (GenStar, China) were as follows: first, predenaturation at 95°C for 2 min, then 95°C for 15 s, 60°C for 30 s, and 72°C for 30 s, for a total of 40 cycles. The primer sequences for PCR amplification were as follows: LUM, forward: 5′-TGAGCTTCAATCAGATAGCCAGAC-3′, reverse: 5′-CACTATCAGCCAGTTCGTTGTGAG-3′; β-actin, forward: 5′-TCCATCATGAAGTGTGACGT-3′, reverse: 5′-GAGCAATGATCTTGATCTTCAT-3′. The relative mRNA expression level was calculated by the 2^−ΔΔCt^ method and standardized to β-actin.

### The Verification of LUM by the GEO Database

A search for the microarray and RNA sequencing that met the requirements from the GEO dataset was performed by using “cancer,” “tumor,” “carcinoma,” or “neoplasm” and “gastric” or “stomach” as the search terms and “*Homo sapiens*” as the qualifier. After excluding the datasets with a small sample size (*n* < 30), a total of 11 datasets (GSE13195, GSE13911, GSE26899, GSE27342, GSE29272, GSE33335, GSE37023, GSE54129, GSE63089, GSE64591, and GSE65801) were downloaded, including 761 GC tissue samples and 475 adjacent nontumor tissue samples as well as LUM expression information (Table 2). Review Manage 5.3 was used to conduct a comprehensive meta-analysis to verify the differences in LUM expression. The combined value was calculated by the standard mean difference (SMD) with a 95% confidence interval (CI). The heterogeneity between the included datasets was evaluated by χ^2^ and *I*^2^ statistical tests. If *P* > 0.05 or *I*^2^ < 50%, the combined effect was calculated by the fixed effect model; otherwise, the random effect model was used (*P* < 0.05 or *I*^2^ > 50%). The results are presented as forest plots.

**Table 2:** Information of selected GEO series dataset.

**GEO datasets**	**Year**	**Country**	**Platform**	**Sample**	***N***
GSE13195	2009	China	GPL5175	GC	25
				Non-GC	25
GSE13911	2008	Italy	GPL570	GC	38
				Non-GC	31
GSE26899	2016	USA	GPL6947	GC	96
				Non-GC	12
GSE27342	2011	USA	GPL5175	GC	80
				Non-GC	80
GSE29272	2013	USA	GPL96	GC	134
				Non-GC	134
GSE33335	2012	China	GPL5175	GC	25
				Non-GC	25
GSE37023	2012	Singapore	GPL96	GC	112
				Non-GC	39
GSE54129	2017	China	GPL570	GC	111
				Non-GC	21
GSE63089	2014	China	GPL5175	GC	45
				Non-GC	45
GSE64591	2015	USA	GPL570	GC	63
				Non-GC	31
GSE65801	2015	China	GPL14550	GC	32
				Non-GC	32

*GEO, Gene Expression Omnibus; GC, gastric cancer*.

### Univariate and Multivariate Cox Regression Analyses

A Cox proportional hazard regression model was used for univariate and multivariate analyses. The hazard ratio and 95% confidence interval were calculated, the independent predictive value of the clinicopathological parameters and LUM expression on survival was quantitatively evaluated, and the independent prognostic effect of LUM on survival was estimated by adjusting for confounding factors. First, we used the Perl programming language to sort and merge the original clinical data, delete the unknown or incomplete parts of clinical information, and match it with the LUM expression data. Finally, we obtained the data of 317 patients, which were analyzed by univariate and multivariate Cox regression. Based on the median LUM expression value, the patients were classified into either the high-LUM expression group or the low-LUM expression group. The data were analyzed and visualized using R software's survival package and survminer package as well as the coxph and ggforest commands.

### Gene Set Enrichment Analysis

GSEA (version 3.0) was used to explore the signaling pathways related to LUM in GC. Gene expression enrichment analysis was carried out between datasets with low or high LUM mRNA expression. The phenotype was determined by the expression level of LUM based on the TCGA database. The annotated gene set was selected (c2.cp.kegg.v6.2.symbols.gmt) as the reference gene set. A total of 1,000 gene sets were arranged in each analysis to determine significantly different pathways. Gene set permutations were performed 1,000 times for each analysis to identify significantly different pathways. The normalized enrichment score (NES), nominal *p*-value, and false discovery rate (FDR) *q*-value indicated the importance of the association between gene sets and pathways.

### Statistical Analysis

The difference in LUM expression between GC tissues and adjacent nontumor tissues was tested by Mann–Whitney U test. The differences in LUM among multiple groups were compared by Kruskal–Wallis test. Chi-square (χ^2^) test was used to evaluate the interrelation between LUM expression and clinicopathological parameters. Kaplan–Meier analysis and log-rank test were used to compare the significant differences in survival rates between the high- and the low-LUM expression groups. A Cox proportional hazard regression model was used for univariate and multivariate survival analysis. All statistical analyses were performed with IBM SPSS statistical software (version 23.0) and R software (version 2.15.3), and *P* < 0.05 was used to determine the significance level.

## Results

### The Difference in LUM Expression in GC

**Figure 1:**
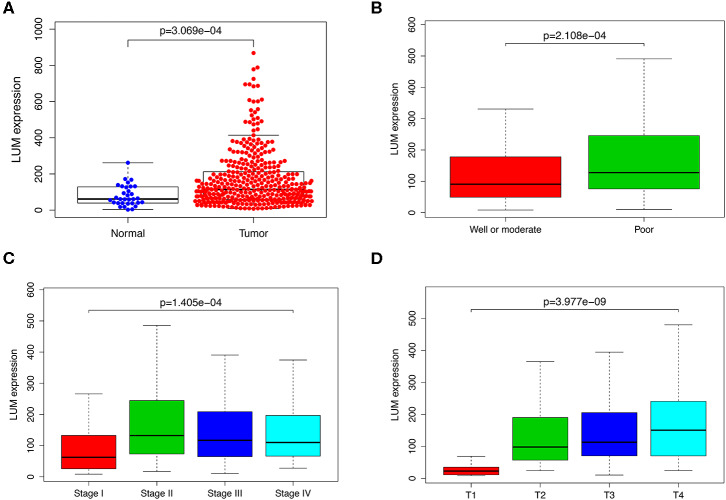
The expression of LUM and its association with clinicopathological parameters based on TCGA data. **(A)** Comparison of LUM expression between gastric cancer tissues and adjacent nontumor tissues. The expression of LUM is grouped by tumor differentiation **(B)**, pathological stage **(C)**, and T stage **(D)**. LUM, lumican; TCGA, The Cancer Genome Atlas.

The LUM expression data at the mRNA level were obtained from 407 tissues (including 375 GC tissues and 32 adjacent nontumor tissues) in the TCGA database. The scatter plot shows the mRNA expression profiles of LUM in GC tissues and adjacent nontumor tissues. As shown in Figure 1A, the expression of LUM was significantly upregulated in GC tissues compared with that in adjacent nontumor tissues (*P* < 0.001). In addition, the expression level of LUM was different in groups classified according to tumor differentiation (*P* < 0.001, Figure 1B), pathological stage (*P* < 0.001, Figure 1C), and T stage (*P* < 0.001, Figure 1D).

### Verification of LUM Upregulation in GC by qRT-PCR and SMD

**Figure 2:**
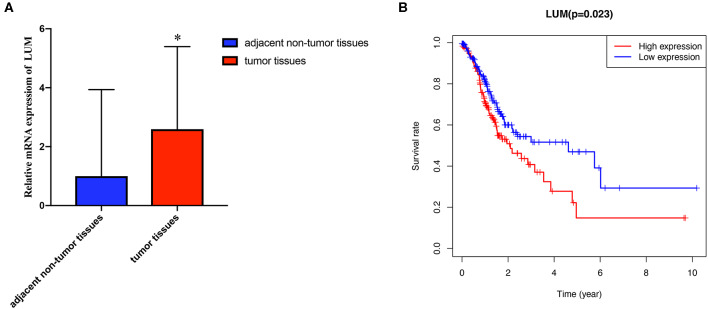
Quantitative real-time polymerase chain reaction analysis and survival analysis. **(A)** qRT-PCR analysis of LUM mRNA expression in 47 pairs of GC tissues and adjacent nontumor tissues. **(B)** Kaplan–Meier curve of the relationship between LUM mRNA expression and the prognosis of GC patients based on TCGA database. qRT-PCR, quantitative real-time polymerase chain reaction analysis; LUM, lumican; GC, gastric cancer; TCGA, The Cancer Genome Atlas. ^*^P < 0.05.

**Figure 3:**
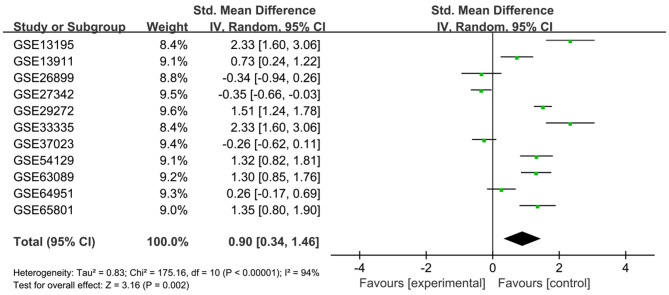
Forest plot of LUM expression data from GEO microarrays. The *I*-squared value was 94%, and the *P* value was <0.001. The pooled SMD of LUM was 0.90 (95% CI, 0.34–1.46) by the random effects model. LUM, lumican; GEO, Gene Expression Omnibus; SMD, standard mean difference; CI, confidence interval.

To verify the difference in LUM expression in the TCGA database, we used qRT-PCR to evaluate the expression of LUM at the transcription level and found that the expression level of LUM mRNA in GC was significantly higher than that in adjacent nontumor tissues (*P* = 0.022, Figure 2A). In addition, a comprehensive meta-analysis of LUM expression data for patients with GC in the GEO dataset was conducted (Table 2). Because the *I*-square value was 94% (*P* < 0.001), the combined SMD of LUM was 0.90 according to the random effects model (95% CI: 0.34–1.46, Figure 3), indicating that LUM was highly expressed in GC.

### High Expression of LUM in GC Is Related to Poor Overall Survival

We evaluated the prognosis of high-LUM expression in GC patients from the TCGA by Kaplan–Meier risk estimates. The results revealed that, compared with the low LUM expression, the high LUM expression was more significantly associated with a poor overall survival (*P* = 0.023, Figure 2B). The median OS of the high-LUM expression group was 12.53 months, while the median OS of the low-LUM expression group was 13.48 months. The 5-year survival rate of patients in the low-LUM expression group (4.89%) was also higher than that of patients in the high-LUM expression group (1.09%).

### The Relationship Between LUM Expression and Clinicopathological Parameters

To further explore the relationship between LUM expression and clinicopathological parameters, the clinical data of 317 patients with GC were downloaded from TCGA database. According to the median expression value, the LUM expression level was divided into a high-LUM expression group and a low-LUM expression group. Table 3 summarizes the correlation between LUM expression level and various clinicopathological parameters in GC patients. The high expression level of LUM was significantly correlated with tumor differentiation (*P* = 0.024) and T stage (*P* = 0.004). Logistic regression analysis showed that the increased expression of LUM in GC was significantly correlated with tumor differentiation (OR = 1.543 for poor vs. well or moderate, *P* = 0.043), pathological stage (OR = 3.149 for stage II vs. stage I, *P* = 0.001; OR = 2.505 for stage III vs. stage I, *P* = 0.007), T classification (OR =13.304 for T2 vs. T1, *P* = 0.014; OR = 18.434 for T3 vs. T1, *P* = 0.005; OR = 30.649 for T4 vs. T1, *P* = 0.001) (Table 4).

**Table 3:** Relationships between lumican (LUM) expression and clinicopathological parameters in GC.

**Clinicopathological parameters**	**LUM expression**		**Total**	***P*-value**
	**High (*n* = 159)**	**Low (*n* = 158)**		
**Age**				
<65 years	71 (53.0)	63 (47.0)	134	0.389
≥65 years	88 (48.1)	95 (51.9)	183	
**Gender**				
Male	99 (50.3)	98 (49.7)	197	0.965
Female	60 (50.0)	60 (50.0)	120	
**Tumor differentiation**				
Well–moderate	48 (41.7)	67 (58.3)	115	**0.024**
Poor	111 (55.0)	91 (45.0)	202	
**Pathological stage**				
I–II	70 (49.0)	73 (51.0)	143	0.697
III–IV	89 (51.1)	85 (48.9)	174	
**T classification**				
T1–T2	28 (35.9)	50 (64.1)	78	**0.004**
T3–T4	131 (54.8)	108 (45.2)	239	
**Lymph node metastasis**				
Negative	53 (53.5)	46 (46.5)	99	0.418
Positive	106 (48.6)	112 (51.4)	218	
**Distant metastasis**				
No	148 (50.2)	147 (49.8)	295	0.988
Yes	11 (50.0)	11 (50.0)	22	

*Bold values indicate P < 0.05. GC, gastric cancer*.

**Table 4:** Lumican (LUM) expression correlated with clinicopathological parameters.

**Clinicopathological parameters**	**Total (*N*)**	**Odds ratio in LUM expression**	***p*-value**
**Age**			
<65 vs. ≥65	371	0.848 (0.561–1.282)	0.435
**Gender**			
Male vs. female	371	0.992 (0.650–1.514)	0.969
**Tumor differentiation**			
Poor vs. well or •moderate	366	1.543 (1.015–2.356)	**0.043**
**Pathological stage**			
StageII vs. stageI	164	3.149 (1.591–6.452)	**0.001**
StageIII vs. stageI	203	2.505 (1.303–4.992)	**0.007**
StageIV vs. stageI	91	2.081 (0.880–5.008)	0.097
**T classification**			
T2 vs. T1	99	13.304 (2.551–245.163)	**0.014**
T3 vs. T1	187	18.434 (3.677–335.410)	**0.005**
T4 vs. T1	119	30.649 (5.953–562.687)	**0.001**
**Lymph node metastasis**			
Positive vs. negative	357	0.870 (0.555–1.363)	0.544
**Distant metastasis**			
Yes vs. no	355	0.923 (0.404–2.094)	0.847

*Bold values indicate P < 0.05*.

### The Effect of LUM Expression on Survival Based on Univariate and Multivariate Analysis

**Figure 4:**
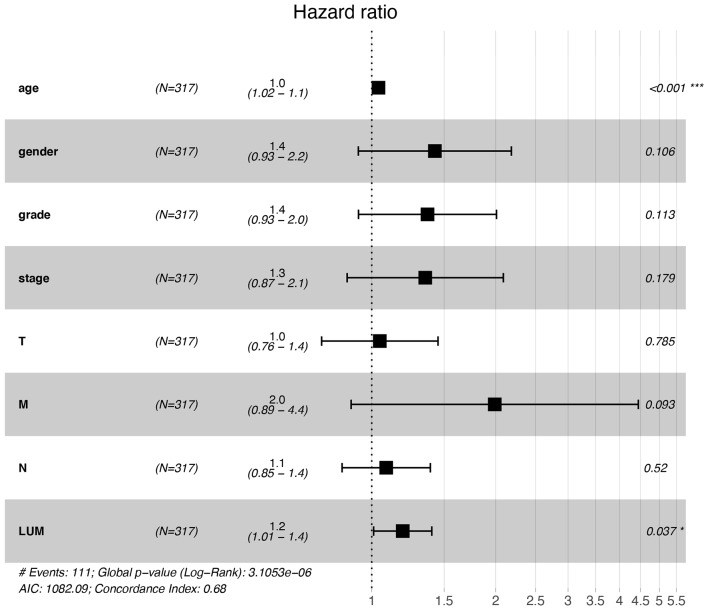
Forest plot for the multivariate Cox proportional hazard regression model. LUM was an independent predictor of poor survival rate (HR, 1.189; 95% CI, 1.011–1.400; *P* = 0.037). LUM, lumican; HR, hazard ratio; CI, confidence interval. ^*^P < 0.05, ^**^P < 0.01, ^***^P < 0.001.

For patients with GC, because survival was significantly correlated with LUM expression, univariate and multivariate analyses were conducted on 317 GC patients according to the Cox proportional hazard regression model to evaluate the impact of LUM expression and other clinicopathological factors on survival. Univariate analysis showed that age (HR, 1.027; 95% CI, 1.008–1.046; *P* = 0.006), pathological stage (HR, 1.535; 95% CI, 1.221–1.931; *P* < 0.001), T stage (HR, 1.298; 95% CI, 1.023–1.645; *P* = 0.032), N stage (HR, 1.267; 95% CI, 1.069–1.502; *P* = 0.006), M stage (HR, 2.048; 95% CI, 1.096–3.827; *P* = 0.025), and LUM expression (HR, 1.219; 95% CI, 1.053–1.412; *P* = 0.008) were important predictors of survival (Table 5). In addition, the expression of LUM and other clinicopathological variables (including age, pathological stage, T stage, N stage, and M stage) were included in the multivariate analysis. The results showed that the high expression of LUM was an important independent predictor of poor overall survival (HR, 1.189; 95% CI, 1.011–1.400; *P* = 0.037) (Table 5, Figure 4).

**Table 5:** Univariate analysis and multivariate analysis of correlation of lumican expression with among GC patients.

**Parameter**	**Univariate analysis**			**Multivariate analysis**		
	**HR**	**95% CI**	***P***	**HR**	**95% CI**	***P***
Age	1.027	1.008–1.046	**0.006**	1.038	1.018–1.059	**0.000**
Gender	1.484	0.980–2.247	0.062	1.424	0.928–2.184	0.106
Grade	1.368	0.947–1.977	0.095	1.366	0.929–2.010	0.113
Pathological stage	1.535	1.221–1.931	**0.000**	1.349	0.871–2.089	0.179
T	1.298	1.023–1.645	**0.032**	1.046	0.756–1.449	0.785
*N*	1.267	1.069–1.502	**0.006**	1.084	0.847–1.388	0.520
M	2.048	1.096–3.827	**0.025**	1.990	0.891–4.445	0.093
LUM	1.219	1.053–1.412	**0.008**	1.189	1.011–1.400	**0.037**

*Bold values indicate P < 0.05. HR, hazard ratio; CI, confidence interval; GC, gastric cancer*.

### Identification of LUM-Related Signaling Pathways by GSEA

**Figure 5:**
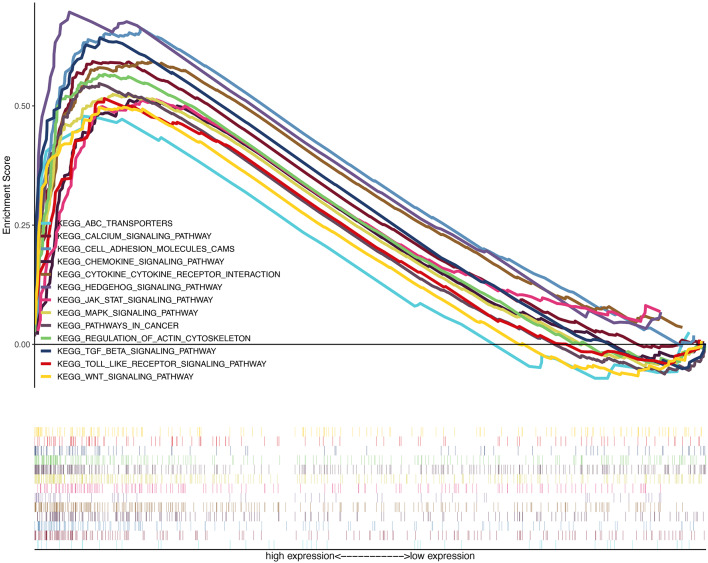
A merged enrichment plot from gene set enrichment analysis including enrichment score and gene sets. The significantly enriched signaling pathways were calcium signaling, hedgehog signaling pathway, cytokine–cytokine receptor interaction, regulation of actin cytoskeleton, TGF-beta signaling, cell adhesion molecules, MAPK signaling, pathways in cancer, JAK-STAT signaling, Wnt signaling, chemokine signaling, Toll-like receptor signaling, and ABC transporters.

On the basis of the TCGA data, we explored the function of LUM and its related signal transduction pathway through GSEA. In view of NES, FDR *q*-value, and nominal *p*-value, significantly enriched signaling pathways were selected. In this study, 14 signaling pathways involved in calcium signaling, hedgehog signaling, cytokine–cytokine receptor interaction, regulation of actin cytoskeleton, TGF-beta signaling, cell adhesion molecules (CAMs), MAPK signaling, pathways in cancer, JAK-STAT signaling, Wnt signaling, chemokine signaling, toll-like receptor signaling, and ABC transporters were differentially enriched in the highly expressed phenotypes of LUM (Table 6, Figure 5, Figure S1).

**Table 6:** Gene sets enriched in the high lumican expression phenotype.

**Gene set name**	**NES**	**NOM *p*-value**	**FDR *q*-value**
KEGG_CALCIUM_SIGNALING_PATHWAY	2.246	0.000	0.000
KEGG_HEDGEHOG_SIGNALING_PATHWAY	2.233	0.000	0.000
KEGG_CYTOKINE_CYTOKINE_RECEPTOR_INTERACTION	2.221	0.000	0.000
KEGG_REGULATION_OF_ACTIN_CYTOSKELETON	2.157	0.000	0.000
KEGG_TGF_BETA_SIGNALING_PATHWAY	2.096	0.000	0.002
KEGG_CELL_ADHESION_MOLECULES_CAMS	2.104	0.000	0.002
KEGG_MAPK_SIGNALING_PATHWAY	2.011	0.000	0.005
KEGG_PATHWAYS_IN_CANCER	2.013	0.000	0.005
KEGG_JAK_STAT_SIGNALING_PATHWAY	1.911	0.000	0.014
KEGG_WNT_SIGNALING_PATHWAY	1.805	0.002	0.029
KEGG_CHEMOKINE_SIGNALING_PATHWAY	1.783	0.012	0.033
KEGG_TOLL_LIKE_RECEPTOR_SIGNALING_PATHWAY	1.758	0.020	0.038
KEGG_ABC_TRANSPORTERS	1.623	0.043	0.076

*NES, normalized enrichment score; NOM, nominal; FDR, false discovery rate*.

## Discussion

The expression of lumican in tumor tissues has been the focus of many studies in recent years. The majority of these studies showed that the expression of lumican was abnormal; the cancer types studied included gastric cancer, colorectal cancer, pancreatic cancer, breast cancer, malignant melanoma, and other malignant tumor tissues. Lumican has two opposite effects on the occurrence and the development of tumors as it can act as an oncogene or a tumor suppressor gene. Some studies have suggested that lumican gene expression in the tumor stroma is higher than that in tumor cells, and as an important intercellular signal molecule in the extracellular matrix, the lumican gene participates in many cellular biological processes and negatively regulates the occurrence and the progression of tumors mainly by affecting the formation of the extracellular matrix or the expression of tumor suppressor genes (22, 28–35). However, it has also been found that its high expression in some tumors is positively correlated with tumor growth and invasion (36–38). Functional enrichment analysis in gastric cancer genomic data showed an upregulation of lumican gene expression with relation to ECM interactions (39). Wang et al. (40) detected the expression of lumican in human gastric cancer, and the results showed that the expression of the lumican gene in gastric cancer tissue was higher than that in noncancerous gastric tissue of the same patient. Therefore, the role of lumican in tumors remains controversial.

In this study, we sought to determine the role of LUM expression in GC progression, especially as a prognostic factor for GC. In addition, we also tried to screen signaling pathways related to LUM in GC to understand the underlying mechanism involved in the regulation of GC development by LUM. First, we analyzed the RNAseq data in the TCGA database and compared the expression of LUM in GC and adjacent nontumor tissues. The expression level of LUM mRNA in GC tissues was significantly higher than that in adjacent nontumor tissues. Then, qRT-PCR and meta-analysis were performed to verify the high expression of LUM in GC, which was consistent with the results of the bioinformatics assay and with the relevant research reports (40). These results suggest that LUM may be an oncogene and play an important role in the progression of GC. In addition, LUM expression levels were different in groups classified according to tumor differentiation, pathological stage, and T stage. The expression of LUM increased with poor differentiation and was upregulated with increasing tumor stage. On this basis, a further analysis of the relationship between LUM expression and clinicopathological parameters showed that the high expression level of LUM was significantly correlated with tumor differentiation and T stage. Takeno et al. (41) found that lumican was significantly upregulated in undifferentiated tumors compared to differentiated tumors in GC. Wang et al. (40) reported that GC lumican overexpression was correlated with late TNM stage and poor survival rate. Collectively, these data indicated that the expression of LUM at the mRNA level is associated with various important clinicopathological parameters, and GC with increased LUM expression is liable to progress to a more advanced stage. In addition, related studies reached similar conclusions at the protein level. Li et al. (42) found that the expression rate of lumican at the protein level in GC tissues was higher than that in adjacent nontumor tissues and further deliberated the close correlations between the lumican protein expression and clinicopathological characteristics including histological type, median overall survival, organ metastasis, and lymph node metastasis. Considering both mRNA and protein expression levels of lumican, Wang et al. (40) quantified the expression level of lumican in GC patients using qRT-PCR and immunohistochemistry and reached an observation that both the mRNA and the protein expression levels of lumican in GC tissues were markedly higher than those in nontumor gastric tissues.

Kaplan–Meier survival analysis showed that the high-LUM expression group had a worse prognosis than the low-LUM expression group. The univariate analysis indicated that high LUM expression was associated with poorer OS. Other clinicopathological parameters, including age, pathological stage, T stage, N stage, and M stage, were correlated with the prognosis of patients with GC. Importantly, we found that LUM was an independent prognostic factor for the overall survival of GC patients and demonstrated its potential to become a biomarker for GC.

The signaling pathway of LUM in GC was analyzed by GSEA. The results showed that the terms calcium signaling pathway, Hedgehog signaling pathway, cytokine–cytokine receptor interaction, regulation of actin cytoskeleton, TGF-beta signaling pathway, CAMs, MAPK signaling pathway, pathways in cancer, JAK-STAT signaling pathway, Wnt signaling pathway, chemokine signaling pathway, Toll-like receptor signaling pathway, and ABC transporters were correlated with the progression of GC. The calcium signaling pathway plays a role in cell cycle progression, survival, apoptosis, migration, and other biological processes (43), and the gene imbalance in this pathway can promote the proliferation, migration, and tumor metastasis of cancer cells (44, 45). The hedgehog signaling pathway affects tumor progression by inducing gene mutations (46), promoting angiogenesis (47), and promoting tumor cell invasion and metastasis (48), and it plays an important role in the evolution of chronic gastritis to gastric cancer (49). Cytokine–cytokine receptor interaction is an important immune signaling pathway because it can regulate the interaction of cytokines to regulate the occurrence and the progression of cancer (50, 51). The actin cytoskeleton is critical for cell migration and cell environment interaction (52). TGF-β can affect cell growth, differentiation, epithelial to mesenchymal transition, and immune regulation (53). The expression level of TGF-β is significantly correlated with tumor size, differentiation, and invasion (54). The CAM pathway is correlated with angiogenesis, invasion, and metastasis of cancer (55, 56). Chemokines are considered to be the main component of cancer-related inflammation, which plays an important role in tumor growth, tumor angiogenesis, and tumor metastasis (57). The tumor-promoting effect of the Toll-like receptor signaling pathway is mainly driven by TLR expressed by tumor cells. TLR stimulation can result in increased cell survival and proliferation or resistance to chemotherapy (58). The overexpression of ABC transporters is related to the resistance of cancer and cancer cells to anticancer drugs (59). The MAPK signaling pathway, the JAK-STAT signaling pathway, and the Wnt signaling pathway are common signaling pathways closely related to cancer, which will not be discussed here. In summary, LUM promotes the development of GC by regulating various signaling pathways.

Our research also has some limitations. First, the clinical information is not perfect, and some important information, such as tumor size, was not provided. Second, there is a lack of specific details, such as surgical treatment and surgical details, which are crucial to the prognosis of patients. Finally, it is impossible to evaluate the protein level and direct mechanism of LUM in GC from TCGA database.

In conclusion, our study first analyzed the TCGA database and found that the expression of LUM in GC tissues is higher than that in adjacent nontumor tissues. The upregulation of LUM is closely correlated with some clinicopathological features of GC, which are related to the occurrence and the development of GC. Importantly, univariate and multivariate survival analyses identified elevated LUM expression in GC as an independent risk factor for shorter OS. In conclusion, we found that the expression level of LUM may be a marker for the diagnosis and the prognosis of GC. In future analyses, other clinical trials will be needed to verify the corresponding results to reveal the prognostic value of LUM in GC.

## Data Availability Statement

Publicly available datasets were analyzed in this study, these can be found in The Cancer Genome Atlas (https://portal.gdc.cancer.gov/); the NCBI Gene Expression Omnibus (GSE13195, GSE13911, GSE26899, GSE27342, GSE29272, GSE33335, GSE37023, GSE54129, GSE63089, GSE64591, GSE65801).

## Ethics Statement

The studies involving human participants were reviewed and approved by the Ethics Committee of Zhongda Hospital, Southeast University. The patients/participants provided their written informed consent to participate in this study.

## Author Contributions

XC designed the overall idea of this study, conceived the experiments, analyzed the data, prepared the figures and tables, and authored the drafts of the paper. XL and XH performed the experiments and collected the data. FJ and YS assisted in downloading the TCGA and GEO dataset. RX and LW did some statistics. PW and XS guided and supervised this study and reviewed the drafts of the paper. All the authors read and approved the final draft.

## Conflict of Interest

The authors declare that the research was conducted in the absence of any commercial or financial relationships that could be construed as a potential conflict of interest.

## Supplementary Material

Figure S1 Enrichment plots from gene set enrichment analysis. **(A)** Calcium signaling pathway, **(B)** hedgehog signaling pathway, **(C)** cytokine–cytokine receptor interaction, **(D)** regulation of actin cytoskeleton, **(E)** TGF-beta signaling pathway, **(F)** cell adhesion molecules, **(G)** MAPK signaling pathway, **(H)** pathways in cancer, **(I)** JAK-STAT signaling pathway, **(J)** Wnt signaling pathway, **(K)** chemokine signaling pathway, **(L)** Toll-like receptor signaling pathway, and **(M)** ABC transporters.
