# Supplementary figures and images for: LUM Expression and Its Prognostic Significance in Gastric Cancer
Source: Front Oncol. 2020 May 15;10:605. doi: 10.3389/fonc.2020.00605 (PMC7242722; doi:10.3389/fonc.2020.00605)

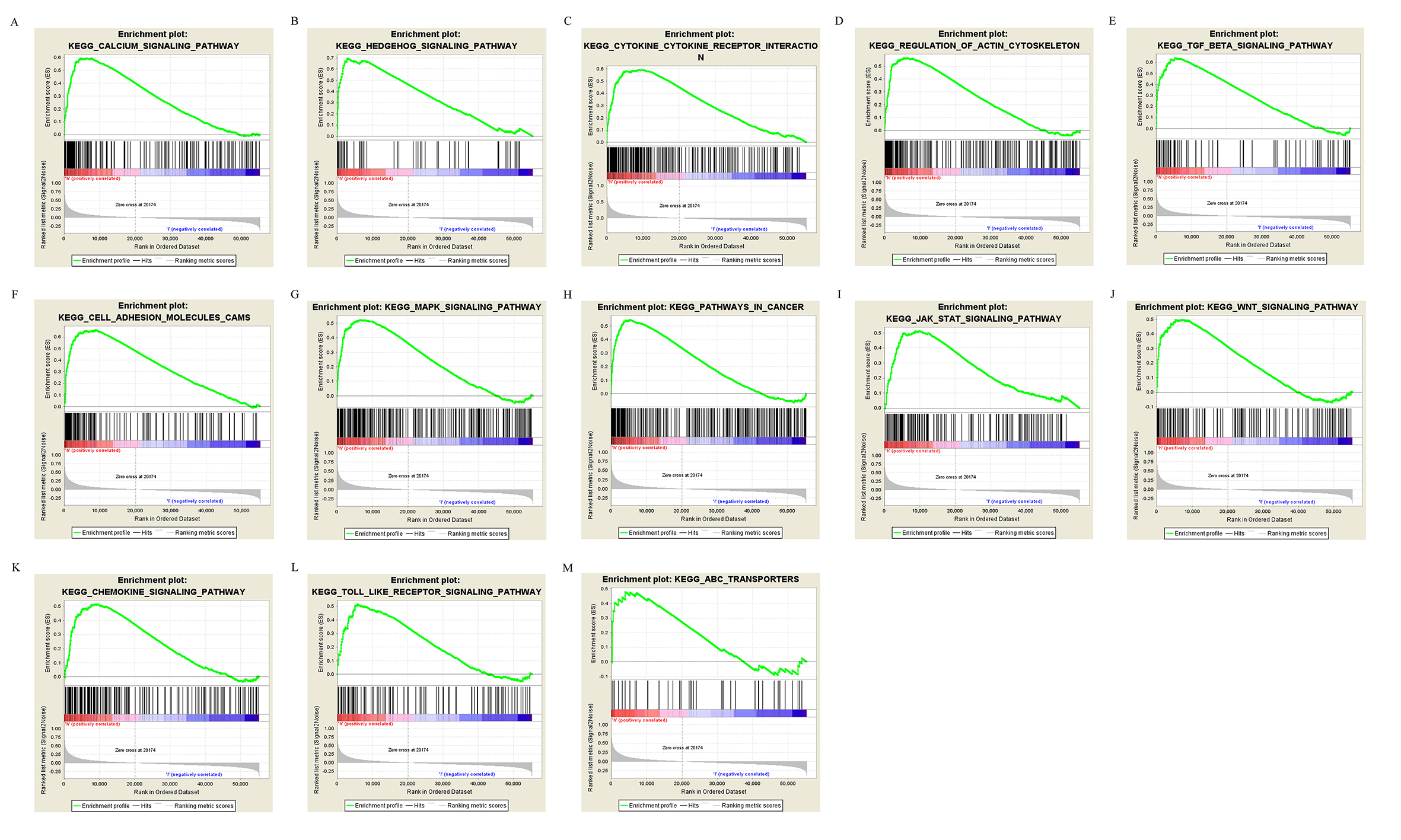

Supplement: Figure S1 — Enrichment plots from gene set enrichment analysis. (A) Calcium signaling pathway, (B) hedgehog signaling pathway, (C) cytokine–cytokine receptor interaction, (D) regulation of actin cytoskeleton, (E) TGF-beta signaling pathway, (F) cell adhesion molecules, (G) MAPK signaling pathway, (H) pathways in cancer, (I) JAK-STAT signaling pathway, (J) Wnt signaling pathway, (K) chemokine signaling pathway, (L) Toll-like receptor signaling pathway, and (M) ABC transporters. [file Image_1.JPEG]
